# Dislocation of the Os Humeri upon the Dorsum Scapulæ, Reduced after the Expiration of Five Weeks

**Published:** 1853-03

**Authors:** 


*Dislocation of the os Humeri upon the Dorsum Scapulæ, reduced after the expiration of five weeks.*—Prof. Paul F. Eve, of the Nashville University, reports a case of this kind in the January number of the Nashville Journal of Medicine and Surgery.

